# Rapid Response and Containment of an NDM-Producing Klebsiella Pneumoniae Outbreak in a Hematology Ward: Case Study from an Italian Hospital

**DOI:** 10.3390/healthcare13121457

**Published:** 2025-06-17

**Authors:** Ilaria Tocco Tussardi, Gloria Stevanin, Livio Montesarchio, Francesca Palladini, Irene Aprili, Emanuela Zandonà, Cristina Tecchio, Stefano Tardivo

**Affiliations:** 1 Department of Diagnostics and Public Health, Section of Hygiene, University of Verona, 37134 Verona, Italy; 2 Hospital Management, University Hospital of Verona, 37126 Verona, Italy; 3 Hematology Unit and Bone Marrow Transplant Center, University Hospital of Verona, 37126 Verona, Italy

**Keywords:** antimicrobial resistance, Klebsiella pneumoniae, New Delhi metallo-beta-lactamase (NDM), infection prevention and control, outbreak

## Abstract

Antimicrobial resistance (AMR) constitutes a critical threat to global public health, with carbapenem-resistant *Enterobacterales* (CRE) presenting significant challenges due to their resistance to last-line antibiotics. Among these, New Delhi metallo-beta-lactamase (NDM)-producing *Klebsiella pneumoniae* (KP) is of particular concern. This study describes an outbreak of NDM-producing KP in the hematology unit of the University Hospital of Verona, Italy. This represents the second reported hospital outbreak of this strain in Italy, and the first to occur within a hematology ward. The outbreak involved four patients, all of whom were identified through active surveillance and microbiological screening. In response, a multidisciplinary team implemented a series of infection prevention and control (IPC) measures, which included enhanced environmental cleaning, strict hand hygiene protocols, patient isolation, and the development of a tailored IPC checklist. The outbreak was effectively contained within three weeks following the identification of the last case. This outcome underscores the importance of rapid and coordinated responses to NDM-producing KP outbreaks. This case study emphasizes the necessity of robust IPC protocols, rapid intervention, and continuous staff education in mitigating the spread of multidrug-resistant pathogens in healthcare settings. It further highlights the urgent need for healthcare systems to be adequately prepared and resilient in addressing the growing threat of AMR.

## 1. Introduction

Antimicrobial resistance (AMR) and multidrug-resistant organisms (MDROs) represent significant global threats to public healthcare systems. These challenges are driven by various factors, including excessive human consumption of antimicrobials, agricultural use of antibiotics, environmental contamination, and healthcare-associated transmissions. Such factors contribute to substantial healthcare costs, including increased morbidity and mortality, particularly among vulnerable populations, as well as expenses related to surveillance, containment programs, and the urgent need for the development of alternative therapeutic agents [1,2].

Recent studies highlighted the growing prevalence of AMR, with the World Health Organization (WHO) predicting that AMR could become a leading cause of death globally by 2050 if not addressed urgently. Furthermore, the economic burden of AMR is expected to reach USD 100 trillion by 2050 due to escalating healthcare costs and diminished workforce productivity [3,4].

A particularly alarming trend is the rise in the global prevalence of carbapenem-resistant *Enterobacterales* (CRE) over the past several decades [5]. According to the European Center for Disease Prevention and Control (ECDC), over a third of European countries reported carbapenem-resistance rates of 25% or higher in *Klebsiella pneumoniae* (KP) isolates in 2021 [6]. The Centers for Disease Control and Prevention (CDC) in the United States has similarly classified CRE as an urgent threat, emphasizing the need for enhanced infection prevention and control (IPC) measures and the development of new antibiotics [7].

Carbapenemase enzymes are categorized into class A (penicillinases), class B (metallo-beta-lactamases, MBL), and class D (oxacillinases), with class B including the high-risk New Delhi metallo-beta-lactamase (NDM) [8,9]. The first detection of NDM-producing KP occurred in 2009 [10], and since then, it has disseminated globally [5,11]. Recent reviews have reported the identification of NDM-producing organisms in over 70 countries, with significant outbreaks in Asia, Europe, and North America [12].

The spread of NDM-producing KP is a significant public health concern due to the near-complete resistance of these strains to available antibiotics, compounded by their transmission in healthcare settings. Of particular concern is the limited availability of effective treatment options for CRE-producing NDM and other metallo-beta-lactamases. Promising agents with activity against carbapenem-resistant strains include cefiderocol and aztreonam/avibactam; however, clinical experience with these agents remains limited, and their use should be guided by susceptibility testing and expert consultation [13]. Hospital outbreaks caused by NDM-producing KP further emphasize the importance of prompt outbreak management, including effective containment strategies to prevent additional patient infections [13,14,15,16,17,18,19,20,21].

This article reports an outbreak of NDM-producing KP in an Italian teaching hospital. To the best of our knowledge, this is the second reported hospital outbreak caused by NDM-producing KP in Italy and the first to occur in a hematology ward. We describe the comprehensive and innovative management approach that led to the rapid and successful containment of the outbreak. The strategy involved the implementation of strict infection control protocols, including active surveillance, environmental controls, and the use of an ad hoc tool to monitor the adherence to IPC practices, demonstrating a model for effective outbreak management in similar healthcare settings.

## 2. Materials and Methods

### 2.1. Setting

The University Hospital of Verona is a tertiary care center serving a population of about 350,000 in northeastern Italy. With 1113 beds, it is the second-largest hospital trust in Italy and ranks fifth nationally in terms of annual admissions (42,500 in 2022). The hospital employs around 6000 staff members, including nearly 1200 medical residents.

The hematology unit, which registered nearly 400 admissions in 2023, functions as a regional referral center for the treatment of acute hematological conditions, with the majority of cases involving hematological malignancies. The unit also includes the Bone Marrow Transplant Center (BMTC). The hematology unit comprises seven double-occupancy rooms and four single rooms, while the BMTC contains seven single, positive-pressure isolation rooms. Although clinical care in the unit and BMTC is managed by separate teams of hematologists, both areas share the same nursing and support staff.

The hospital conducts active surveillance for pathogens of epidemiological concern, including carbapenem-resistant CRE. Rectal swab samples were collected upon patient admission to the ward. These swabs are used to screen patients for colonization by carbapenem-resistant CRE or other MDROs of particular concern. Microbiological evaluation includes culture-based isolation followed by resistance profiling, which is performed using molecular techniques to detect resistance genes. Strains isolated from clinical specimens—such as respiratory, blood, or urinary samples—collected due to suspected infections are subjected to antimicrobial susceptibility testing according to EUCAST guidelines, in order to determine resistance patterns to key antibiotic agents. Additionally, molecular techniques such as multilocus sequence typing (MLST) can be employed to characterize hypervirulent or high-risk clones, which are of particular epidemiological relevance at both national and European levels. The hospital has identified a list of priority MDROs for targeted surveillance, which includes the following: multidrug-resistant *Acinetobacter baumannii* (MDR-A. baumannii), non-fermenting Gram-negative bacilli (e.g., *Pseudomonas* spp., *Burkholderia* spp., *Stenotrophomonas maltophilia*) with MDR or XDR profiles, *Enterobacterales* resistant to third-generation cephalosporins (cefotaxime, ceftriaxone, ceftazidime), vancomycin-resistant *Enterococcus faecalis* and *Enterococcus faecium* (VRE), methicillin-resistant *Staphylococcus aureus* (MRSA), and MRSA strains with reduced susceptibility to glycopeptides. In addition to the MDROs listed above, the regional surveillance system also includes *Aspergillus* spp. in immunocompromised patients and toxin-producing *Clostridium difficile*. Patients admitted to the hematology unit and the BMTC undergo additional screening via rectal swabs every seven days.

### 2.2. Outbreak Recognition

A Ukrainian patient (Patient 1, P1) was admitted to the hematology unit on 5 April 2022. She had no history of hospitalization within the preceding 12 months. After 15 days of hospitalization, a routine rectal swab returned positive for NDM-producing KP. Following this finding, an alert was issued and all patients in the ward were placed under contact precautions. On 5 May, the patient was transferred to a general medical ward to continue treatment for her hematological condition. She was readmitted to the hematology unit on 3 August, with known colonization, and subsequently transferred to a bed in the BMTC on 12 August to receive treatment in preparation for donor stem cells transplantation. She remained in the BMTC until 17 August, when she was transferred to the intensive care unit due to the deterioration of her general conditions. The patient died on 18 August.

Seven days after P1’s admission to the BMTC, on 19 August, a second patient (P2) hospitalized in the same unit tested positive for carbapenem-resistant KP (CR-KP) through rectal screening. The patient exhibited no clinical signs of infection.

Microbiological specimens—including gastrointestinal, urinary, blood, and respiratory samples—were collected from all patients from both the hematology ward and the BMTC, to assess other infections caused by NDM-producing KP. All patients who tested positive for this organism were subject to continued monitoring, with follow-up specimen collection carried out in the subsequent weeks to track colonization or infection status. A third patient (P3) was identified as positive via rectal swab on 2 September, followed by a fourth patient (P4), who tested positive on both rectal and pharyngeal swabs on 9 September. The anatomical sites of isolation for NDM-producing KP in the four affected patients are summarized in Table 1.

Antimicrobial susceptibility test was carried out on respiratory samples from P1. The susceptibility profile is reported in Table 2. These results confirm a multidrug-resistant (MDR) phenotype, consistent with the presence of an NDM-producing strain.

## 3. Results

### 3.1. Control of the Outbreak

A multidisciplinary team was promptly assembled following the detection of the second case to coordinate the outbreak response. On 23 August 2022, rectal swabs were collected from all patients in the hematology unit and the BMTC, with subsequent screening conducted weekly. In addition, on-site inspections were carried out, and microbiological sampling of environmental surfaces and medical equipment was performed. Environmental sampling focused on surfaces, particularly high-touch areas within patient rooms. Microbiological environmental sampling was performed on both surfaces and medical equipment, including the patient zone and the surrounding care area. IPC measures were reinforced among healthcare personnel, and environmental hygiene protocols were intensified, including deep cleaning of high-risk areas (e.g., toilets) and enhancement of routine cleaning practices.

To identify IPC strategies commonly recommended for managing outbreaks of NDM-producing KP in hospital settings, a scoping review of the literature was conducted. The review included searches in PubMed and Web of Science, as well as relevant documents from authoritative institutional websites. Eligible documents reported IPC strategies, best practices, or protocols specifically addressing outbreaks caused by NDM-producing KP. Publications focusing on other pathogens or unrelated emergency responses were excluded.

From the review, seven key areas of action were identified as essential for institutional preparedness and resilience during NDM-producing KP outbreaks. For each area, specific actions (referred to as ‘items’) were compiled into an ad hoc checklist developed for outbreak management. The checklist comprises 60 items grouped under the following seven domains:

1. General measures for personnel management during an epidemic;
2. Measures to ensure an environment conducive to IPC;
3. Identification of colonized or infected patients and communication with relevant hospital committees;
4. Case isolation procedures;
5. Alternative measures when single-room isolation is not feasible;
6. Standard precautions and pathogen-specific transmission control measures;
7. Appropriate use of antimicrobials and invasive devices to optimize patient outcomes and reduce the risk of resistance.

Each checklist item was formulated as a question, allowing users to indicate whether the measure was currently in place and operational within the facility. An additional column enabled the reporting of any institutional documents (e.g., protocols, guidelines) supporting the measure. This tool was designed to assess the current state of IPC implementation and to guide targeted interventions. The complete checklist and the results of its application in this outbreak are provided in Table 3.

### 3.2. Follow Up

All environmental samples tested negative for NDM-producing KP. Following the discharge of the last patient from the BMTC, no new cases of CRE were identified in the subsequent three weeks, at which point the alert was lifted. Additional training sessions on hand hygiene and transmission-based precautions were scheduled and conducted in the months following the outbreak. Routine surveillance over the 12 months following the resolution of the outbreak revealed no further cases of NDM-producing KP.

## 4. Discussion

Identifying the origin of NDM-producing KP in outbreak settings is often complex. Transmission commonly occurs through direct patient contact—particularly in shared-room settings—and indirectly via contaminated environments and equipment [22,23]. The increasing movement of refugees and migrants, some of whom may be colonized with MDRO, further complicates the epidemiological landscape. In our case, the involvement of a Ukrainian patient highlights how geopolitical events can influence pathogen dissemination [24,25,26].

The effective management of NDM-producing KP outbreaks requires the prompt implementation of comprehensive IPC strategies, including contact precautions, hand hygiene, environmental decontamination, patient isolation, and active surveillance [25]. In some situations, limiting admissions may be necessary to contain the outbreak [26].

Environmental investigations are essential to identify potential reservoirs and tailor interventions to disrupt transmission routes [27,28]. The effectiveness of such measures is supported by previous reports, including those from Tuscany (2018–2019) and Pisa (2020), where outbreaks were linked to clones previously reported in the Middle East [29,30,31]. These patterns align with the broader trend of geographic expansion of NDM-producing KP across Europe [32,33].

In the outbreak described, inter-human transmission was likely the predominant route. Although environmental sampling yielded no positive results and IPC measures were reportedly followed, several contextual factors may have contributed to the spread. These include the presence of students in the BMTC, cross-coverage by staff between BMTC and other wards, and the seasonal use of temporary personnel from wards with lower IPC standards. These staff may not have been fully trained in HAI prevention. However, the influence of uncontrolled variables or random events cannot be excluded. Notably, the first patient’s rectal swab at admission was negative, indicating that colonization occurred during hospitalization. This finding reinforces the critical need to evaluate and strengthen routine IPC measures within the healthcare setting.

The introduction of a structured, outbreak-specific checklist proved instrumental in organizing the response. The tool enabled systematic monitoring across key domains such as staff management, environmental safety, patient tracking, isolation procedures, IPC practices, and antimicrobial stewardship. Regular interdisciplinary meetings facilitated prompt assessment and action. Furthermore, integrating similar checklists into routine IPC monitoring may enhance institutional preparedness and resilience for future outbreaks.

### Limitations and Future Directions

The main limitation of this study is the absence of molecular typing or whole-genome sequencing of the NDM-producing KP isolates, which would have provided more definitive evidence of transmission dynamics. Despite this, the epidemiological links observed strongly support the hypothesis of inter-human spread. Future investigations should incorporate genomic analyses to confirm transmission pathways and support more targeted containment strategies.

## 5. Conclusions

The containment of NDM-producing Enterobacterales in healthcare settings demands a robust, multidisciplinary approach that integrates strict IPC measures, rapid response capabilities, and continuous staff training. Structured tools, such as the customized checklist described, can enhance outbreak management and institutional coordination. To counter the growing threat of antimicrobial resistance, hospitals must prioritize preparedness, invest in preventive infrastructures, and foster a culture of IPC excellence.

## Figures and Tables

**Table 1:** Isolations of NDM-producing *Klebsiella pneumoniae* (KP) in four patients.

Patient	Admission (Date, Unit)	First Isolation of NDM-Producing KP (Date, Sample)	Subsequent Isolations (Sample)
1	5 April, Hematology 3 August, BMTC	21 April, rectal swab	Bronchoalveolar lavage Bronchoaspirate Blood culture
2	5 August, BMTC	19 August, rectal swab	Urine culture
3	23 August, BMTC	2 September, rectal swab	—
4	28 July, BMTC	9 September, rectal and pharyngeal swabs	—

**Table 2:** Antimicrobial susceptibility profile of *Klebsiella pneumoniae* strains isolated from respiratory samples from Patient 1. S = Susceptible; I = Intermediate; R = Resistant (SIR).

Antibiotic	SIR
Amikacin	R
Amoxicillin/Clavulanic acid	R
Cefepime	R
Cefotaxime	R
Ceftazidime	R
Ciprofloxacin	R
Ertapenem	R
Fosfomycin	R
Genamicin	R
Imipenem	R
Piperacillin/Tazobactam	R
Trimethoprim/Sulfamethoxazole	R

**Table 3:** IPC checklist for managing NDM-Producing *Klebsiella pneumoniae* (KP) in hospital settings.

Area/Item	Note/Action
**General measures for personnel management during an epidemic outbreak**	
Procedure to control the risk of NDM-producing KP spread?	Present, applied
Professional in charge of IPC measures, especially for MRDO?	Present (1 physician and 2 nurses)
Risk assessment for the prevention and control of NDM KP infections?	Conducted
Rectal swabs to detect MDRO performed at admission and weekly?	Yes
Activation of disinfection and decontamination procedures? Supervision?	Yes, supervised by the hygiene service
Staff provided with regular information, training, and supervision regarding IPC measures, standard and specific precautions?	Yes, by the hygiene service
Protocol for sharing information about colonization/infection by NDM KP at transfer/discharge?	Present, applied
In case of a suspected outbreak, was the unit identified as high risk and early isolation measures activated?	Yes
NDM-producing KP-positive patient adequately informed about the modes of transmission?	Yes, by the physician in charge
Caregiver of the NDM-producing KP-positive patient adequately informed and trained?	Yes
Procedures for the transportation of a positive patient within the hospital applied correctly?	Yes
Information to minimize risk provided at discharge of a positive patient?	Yes, colonization status highlighted in the discharge letter
Operators transporting a positive patient adequately informed?	Yes
Restrictions of access to the ward during the alarm period (e.g., limiting the presence of non-essential personnel)?	Yes, access to the ward for students was suspended
**Measures to ensure the adequacy of the hospital environment for IPC**	
Procedures for cleaning, disinfection and decontamination of equipment/objects in the event of an outbreak by NDM-producing KP?	Present, applied
Room of the NDM-producing KP-positive patient cleaned according to the procedure?	Yes
Bathroom of the NDM-producing KP positive patient cleaned twice a day?	Yes
Additional cleaning procedures carried out during the hospital stay and on discharge of the positive patient?	Yes
Environmental swabs carried out to check the effectiveness of the cleaning actions?	No
Do hospital cleaning staff understand and correctly implement cleaning and disinfection procedures?	Yes. Inspections carried out by the hygiene service
Cleaning procedures foreseen in case of NDM-producing KP outbreaks recorded?	Yes, cleaning interventions recorded
**Identification of positive patients and communication to the responsible committees**	
Did active contact surveillance include screening of all contacts of the source and secondary cases?	Yes
Screening carried out according to the hospital protocol?	Yes
Screening performed on all incoming patients?	Yes
Screening repeated every 7 days for 3 weeks after the discharge of the last patient in the outbreak?	Yes
In the suspect of an outbreak, were the Epidemiological Observatory, the Infection Control Officer and the Microbiology Service notified?	Yes
Outbreak Management Team activated?	Yes
Outbreak Management Team conducted an appropriate epidemiological investigation?	Yes
Surveillance data collected shared with the personnel of the unit interested by the outbreak?	Yes
**Isolation of a case**	
Positive patient isolated in a single room?	Yes, single rooms were available
Signs indicating isolation of the patient?	Yes
Dedicated staff available for the positive patient?	Not feasible based of the unit’s staffing. Ensured continuity of specialized nursing and medical care
In the presence of multiple cases, dedicated staff used of designed nurses identified?	Not feasible based of the unit’s staffing. Ensured continuity of specialized nursing and medical care
Additional measures implemented to reduce the risk of pathogen transmission?	Increased awareness and training of the staff
**Procedures to follow when single-room isolation is not possible**	
Cohorting of positive patients conducted?	Yes
Additional isolation measures activated in shared rooms (functional isolation)?	Yes
Room shared only with positive patients or with low-risk negative patients?	Yes
Carts with dedicated PPE and dedicated waste bin placed next to the patient’s bed?	Yes
Medical devices/PPE removed before exiting the room?	Yes
**Standard precautions and specific measures for controlling infection transmission**	
Hand hygiene practices followed by healthcare workers?	Yes
Adherence to hand hygiene procedure evaluated and how?	Yes, by direct observation from the Hygiene service
Training courses on hand hygiene organized for all healthcare personnel?	Yes, training courses for healthcare staff conducted in 2021 and 2022
Consumption of alcohol gel evaluated?	Yes
Healthcare workers trained on the use of PPE?	Yes, specific training received
Correct use of PPE evaluated and how?	Yes, by direct observation from the Hygiene service
Regular cleaning and disinfection of the environment, items and reusable medical devices (reconditioning/reprocessing) carried out?	Yes
How were cleaning and disinfection evaluated?	By the Hygiene service
Dedicated equipment for positive patients? (e.g., stethoscope, blood pressure cuff, glucometer, oximeter, thermometer, etc.)	Yes
Proper management and disposal of linen and waste?	Yes
Were contact precautions implemented as required?	Yes
How were contact precautions evaluated?	By direct observation
**Ensuring appropriate use of antimicrobials and invasive devices to optimize patient outcome and reduce the risk of adverse events and the emergence of MDRO**	
Was antibiotic therapy evaluated with the infectious disease consultant?	Yes
Was appropriate use of invasive devices conducted?	Yes

## Data Availability

Additional data supporting the findings of this study are available from the corresponding author upon reasonable request.

## Abbreviations

The following abbreviations are used in this manuscript:

NDM	New Delhi metallo-beta-lactamase
KP	Klebsiella pneumoniae
MDRO	Multi-drug resistant organism
IPC	Infection prevention and control
HAI	Healthcare-associated infection
BMTC	Bone Marrow Transplant Center
